# Prenatal Ultrasound Diagnosis of Complete Pentalogy of Cantrell: A Case Report

**DOI:** 10.7759/cureus.107713

**Published:** 2026-04-25

**Authors:** Divya Jain, Shruti M Mundada, Manali Mundada, Lochan Sai Ganesh Bandreddi, Tejasree Barre

**Affiliations:** 1 Radiology, Gandhi Medical College and Hospital, Secunderabad, IND; 2 Internal Medicine, Gandhi Medical College and Hospital, Secunderabad, IND; 3 General Medicine, Osmania Medical College, Hyderabad, IND; 4 Medicine, Gandhi Medical College and Hospital, Secunderabad, IND

**Keywords:** antenatal diagnosis, congenital anomaly, cystic hygroma, ectopia cordis, fetal autopsy, omphalocele, pentalogy of cantrell, prenatal ultrasound, thoracoabdominal wall defect, toyama classification

## Abstract

Pentalogy of Cantrell is a rare congenital anomaly characterized by a spectrum of midline thoracoabdominal defects involving the abdominal wall, sternum, diaphragm, pericardium, and heart. We report a case of a 32-year-old primigravida diagnosed during a routine second-trimester anomaly scan with a fetus demonstrating a large supraumbilical abdominal wall defect with herniated viscera, ectopia cordis, and associated diaphragmatic and sternal defects. Additional findings included cystic hygroma and kyphoscoliosis. The constellation of findings fulfilled Toyama Class I criteria for complete pentalogy of Cantrell. Following multidisciplinary counseling regarding the poor prognosis, the pregnancy was medically terminated. Post-termination fetal examination confirmed the prenatal imaging findings and classical pentad. This case highlights the critical role of targeted antenatal ultrasound in early diagnosis, differentiation from similar anomalies, and guiding timely clinical decision-making.

## Introduction

Pentalogy of Cantrell (PoC) is a rare congenital malformation complex characterized by a spectrum of midline defects involving the anterior abdominal wall, sternum, diaphragm, pericardium, and heart [1,2]. First described by Cantrell et al. in 1958, the condition demonstrates variable clinical presentation depending on the extent of thoracoabdominal and intracardiac involvement [1,3]. The complete form, classified as Toyama Class I, includes all five components of the classical pentad and is associated with a particularly poor prognosis, especially in the presence of ectopia cordis [3,4].

The estimated prevalence of PoC ranges from approximately one in 65,000 to 200,000 live births, underscoring its rarity in clinical practice [5,6]. Although most cases occur sporadically, occasional familial occurrences have been reported, suggesting possible genetic or developmental influences [3]. Ectopia cordis, one of the most severe manifestations of PoC, is associated with poor perinatal survival due to complex cardiac and thoracoabdominal defects [4,7,8]. The embryological basis of PoC is believed to involve failure of ventral mesodermal development and defective midline fusion during early gestation, particularly between days 14 and 18 [9,10].

Prenatal imaging plays a crucial role in the diagnosis of PoC. Ultrasound remains the primary modality and can identify key features such as ectopia cordis and anterior abdominal wall defects during routine antenatal evaluation [11]. Early recognition of these findings is essential for accurate diagnosis, differentiation from other congenital anomalies, and appropriate counseling regarding prognosis and pregnancy management. To our knowledge, reports describing second-trimester diagnosis of complete pentalogy of Cantrell with post-termination anatomical correlation remain limited, highlighting the importance of detailed prenatal imaging in confirming classical features of the syndrome.

## Case presentation

**Figure 1:**
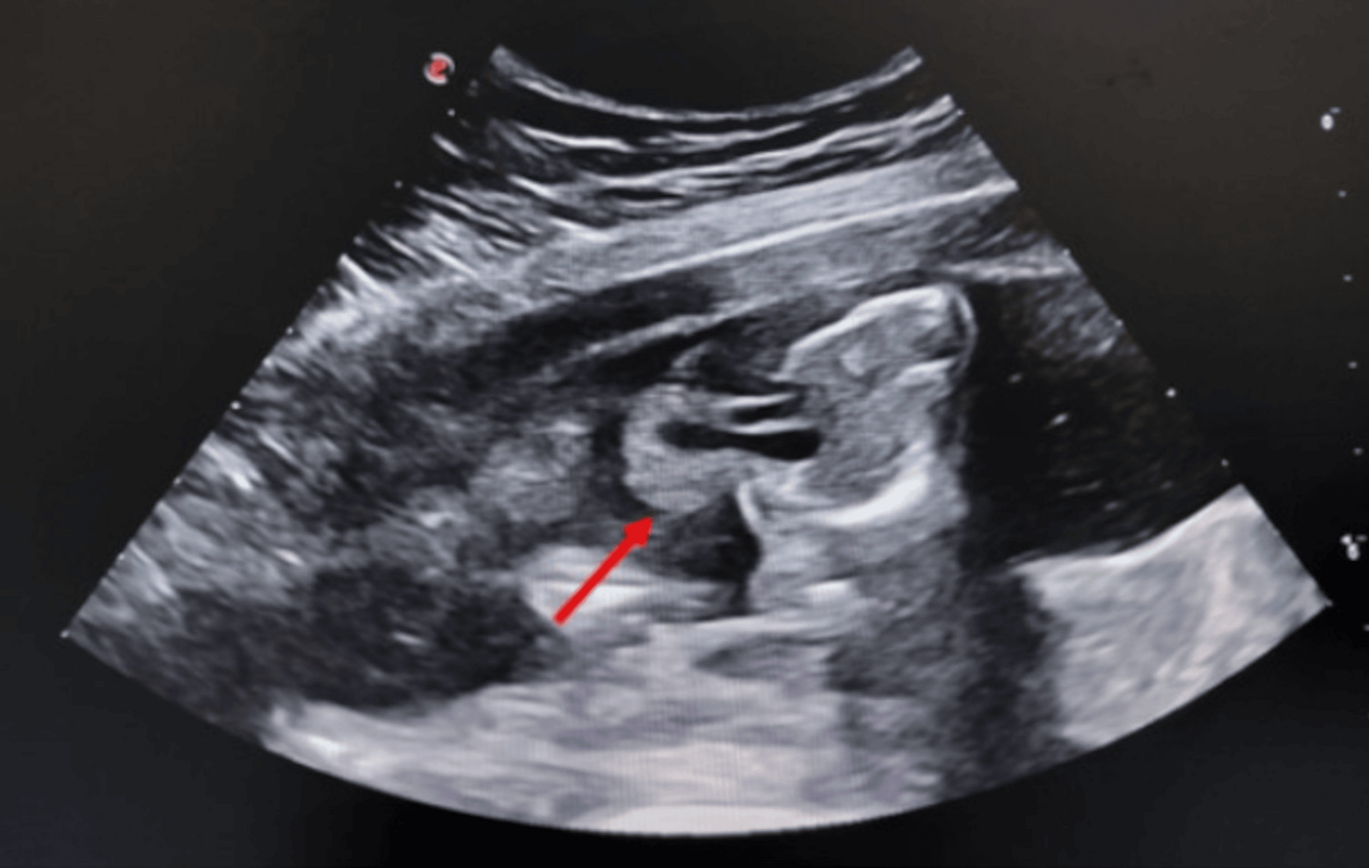
Antenatal ultrasound showing thoracic wall defect with ectopia cordis. B-mode antenatal ultrasound, axial plane at the level of the fetal chest demonstrating a midline anterior thoracic wall defect with partial ectopia cordis (red arrow).

A 32-year-old primigravida at 22 weeks+three days of gestation presented for a routine second-trimester anomaly scan. The pregnancy was spontaneous and had been uneventful until the time of evaluation. There was no history of teratogen exposure, febrile illness, radiation exposure, or consanguinity. No prior first-trimester anomaly scan records were available. Ultrasound examination demonstrated a single live male fetus in breech presentation with a fetal heart rate of 126 beats per minute. Mild oligohydramnios was noted, with an amniotic fluid index of approximately 5-6 cm. Evaluation of the thorax revealed a large anterior thoracic wall defect with extrathoracic herniation of the heart enclosed within a membranous sac, consistent with ectopia cordis (Figure 1).

**Figure 2:**
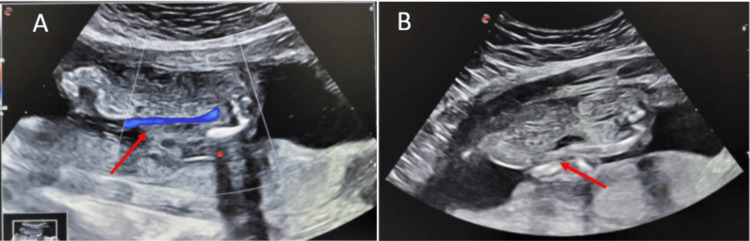
Prenatal ultrasound demonstrating omphalocele and umbilical cord abnormality. (A, B) B-mode antenatal ultrasound at the level of the fetal abdomen showing a supraumbilical anterior abdominal wall defect with a large omphalocele (red arrows). Color Doppler demonstrates a single umbilical artery (blue signal).

The anterior diaphragm showed discontinuity, suggestive of an associated diaphragmatic defect. Abdominal assessment demonstrated a supraumbilical anterior abdominal wall defect with herniation of abdominal viscera covered by a membrane, consistent with a large omphalocele (Figures 2A, 2B).

**Figure 3:**
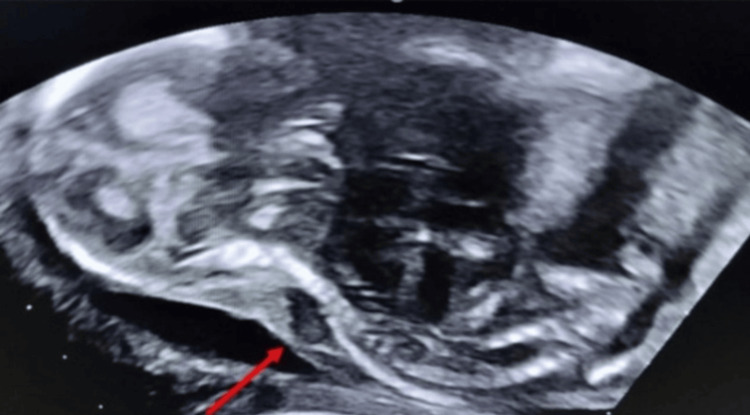
Prenatal ultrasound showing associated skeletal and neck anomalies. B-mode antenatal ultrasound, sagittal view, demonstrating cystic hygroma at the level of the fetal neck and associated kyphoscoliosis of the spine (red arrow).

The umbilical cord was seen inserting at the base of the sac, and color Doppler evaluation demonstrated a single umbilical artery. The placenta was normally located and appeared unremarkable. Additional findings included a thickened nuchal fold with a cystic component suggestive of cystic hygroma, along with abnormal curvature of the spine consistent with kyphoscoliosis (Figure 3).

Fetal parts were contained within the amniotic cavity, aiding in differentiation from the limb-body wall complex, which is typically characterized by abnormal placentation, a short or absent umbilical cord, and fetal parts located outside the amniotic cavity [12]. Based on the constellation of findings, a diagnosis of complete pentalogy of Cantrell (Toyama Class I) was made. Following multidisciplinary counseling involving obstetrics, radiology, and fetal medicine teams, the family was informed about the poor prognosis associated with this condition, particularly in the presence of ectopia cordis. After informed consent, medical termination of pregnancy was performed.

**Figure 4:**
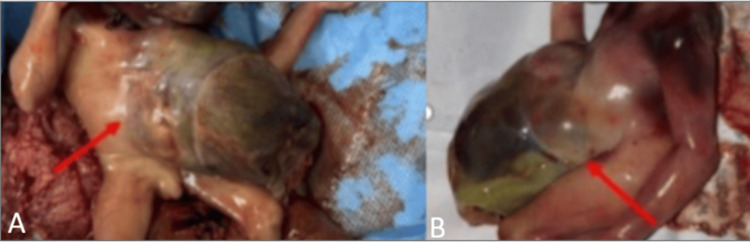
Post-termination gross examination confirming pentalogy of Cantrell. Post-termination specimen photographs in anteroposterior (A) and sagittal (B) views demonstrating a supraumbilical midline anterior thoracoabdominal wall defect with herniation of cardiac and abdominal viscera covered by a membranous sac, consistent with complete pentalogy of Cantrell (Toyama Class I).

Post-termination gross fetal examination confirmed the prenatal findings, including ectopia cordis, a supraumbilical omphalocele containing herniated liver and bowel loops, an anterior diaphragmatic defect, absence of the lower sternum, and pericardial discontinuity, corroborating the diagnosis (Figures 4A, 4B).

## Discussion

Pentalogy of Cantrell is a rare congenital malformation complex characterized by defects involving the anterior abdominal wall, sternum, diaphragm, pericardium, and heart. The syndrome is believed to result from abnormal embryologic development during early gestation, particularly between days 14 and 18, when ventral mesodermal migration and midline fusion occur [13,14]. Disruption of this process can lead to defective formation of thoracoabdominal structures and the characteristic constellation of anomalies observed in this condition.

Toyama’s classification system remains widely used to categorize pentalogy of Cantrell based on the completeness of defects [3]. Class I represents the complete form with all five classical components, Class II includes cases with four defects, and Class III represents incomplete variants with variable combinations. The present case demonstrated all five components of the classical pentad and is therefore consistent with Toyama Class I, which is typically associated with a poorer prognosis.

Prenatal imaging plays a central role in the diagnosis of pentalogy of Cantrell. Ultrasonography is the primary diagnostic modality and can demonstrate characteristic findings such as ectopia cordis and a supraumbilical abdominal wall defect, which together strongly suggest the diagnosis [11,15]. In some cases, these abnormalities may be detected as early as the first trimester; however, the complete spectrum of anomalies is more commonly identified during the second-trimester anomaly scan. In the present case, antenatal ultrasound demonstrated ectopia cordis, a large omphalocele, diaphragmatic discontinuity, and associated structural abnormalities, allowing a confident prenatal diagnosis.

Differentiation from other anterior abdominal wall defects is essential for appropriate counseling and management. Conditions such as gastroschisis, limb-body wall complex, and omphalocele, exstrophy of cloaca, imperforate anus, and spinal defects (OEIS) complex may demonstrate overlapping imaging features [16]. Limb-body wall complex typically presents with abnormal placental attachment, a short or absent umbilical cord, and fetal parts located outside the amniotic cavity. In contrast, pentalogy of Cantrell usually demonstrates a normally positioned placenta, umbilical cord insertion at the base of the omphalocele, and fetal parts contained within the amniotic cavity. The findings in this case were consistent with these distinguishing features, supporting the diagnosis of pentalogy of Cantrell.

The prognosis of pentalogy of Cantrell largely depends on the severity of intracardiac anomalies and the presence of ectopia cordis. Complete forms are associated with high perinatal mortality despite advances in neonatal care and surgical techniques [2,13]. Although incomplete variants without major cardiac involvement may be amenable to staged surgical repair, complete Toyama Class I cases generally carry a poor prognosis due to the complexity of thoracoabdominal defects and associated cardiopulmonary compromise.

Recent studies have explored possible genetic and molecular mechanisms underlying the syndrome. Variations in genes such as ALDH1A2, which plays a role in retinoic acid signaling and mesodermal development, have been reported to be associated with this condition [17]. Additionally, chromosomal abnormalities, including 1p36 deletion, have been described in cases with anterior body wall defects [18]. However, most reported cases appear to occur sporadically, suggesting a multifactorial etiology.

This case highlights the importance of meticulous fetal anatomical evaluation during routine antenatal ultrasound. Early recognition of characteristic imaging features enables accurate diagnosis, differentiation from similar anomalies, and timely multidisciplinary counseling regarding prognosis and pregnancy management.

## Conclusions

Prenatal ultrasound plays a crucial role in the early diagnosis of pentalogy of Cantrell. The presence of ectopia cordis in association with a supraumbilical abdominal wall defect should prompt careful evaluation for additional anomalies and differentiation from other anterior abdominal wall defects. Early recognition of these characteristic findings enables accurate diagnosis, facilitates multidisciplinary counseling, and supports informed decision-making regarding pregnancy management.
